# Identification and Functional Analyses of Host Proteins Interacting with the P3a Protein of Brassica Yellows Virus

**DOI:** 10.3390/biology12020202

**Published:** 2023-01-28

**Authors:** Si-Yuan Liu, Deng-Pan Zuo, Zong-Ying Zhang, Ying Wang, Cheng-Gui Han

**Affiliations:** Ministry of Agriculture and Rural Affairs Key Laboratory of Pest Monitoring and Green Management, College of Plant Protection, China Agricultural University, Beijing 100193, China

**Keywords:** poleroviruses, P3a, membrane yeast two-hybrid, interaction

## Abstract

**Simple Summary:**

A newly identified non-AUG-initiated ORF, ORF3a, encoded by members of the genus *Polerovirus*, is required for long-distance movement in plants. However, its functions in host protein interactions still remain unclear. Here, we systemically investigated Brassica yellows virus (BrYV)-P3a interacting proteins in plants. In total, 138 genes with annotations were obtained. Furthermore, *Arabidopsis thaliana* purine permease 14, glucosinolate transporter 1, and nitrate transporter 1.7 were verified to interact with P3a in vivo and were downregulated in response to BrYV during the late stages of viral infection. We used *pup14*, *gtr1*, and *nrt1.7*, the T-DNA insertion mutants, to preliminarily characterize their roles in the BrYV infection process.

**Abstract:**

Viruses are obligate parasites that only undergo genomic replication in their host organisms. ORF3a, a newly identified non-AUG-initiated ORF encoded by members of the genus *Polerovirus*, is required for long-distance movement in plants. However, its interactions with host proteins still remain unclear. Here, we used Brassica yellows virus (BrYV)-P3a as bait to screen a plant split-ubiquitin-based membrane yeast two-hybrid (MYTH) cDNA library to explain the functional role of P3a in viral infections. In total, 138 genes with annotations were obtained. Bioinformatics analyses revealed that the genes from carbon fixation in photosynthetic, photosynthesis pathways, and MAPK signaling were affected. Furthermore, *Arabidopsis thaliana* purine permease 14 (AtPUP14), glucosinolate transporter 1 (AtGTR1), and nitrate transporter 1.7 (AtNRT1.7) were verified to interact with P3a in vivo. P3a and these three interacting proteins mainly co-localized in the cytoplasm. Expression levels of *AtPUP14*, *AtGTR1*, and *AtNRT1.7* were significantly reduced in response to BrYV during the late stages of viral infection. In addition, we characterized the roles of AtPUP14, AtGTR1, and AtNRT1.7 in BrYV infection in *A. thaliana* using T-DNA insertion mutants, and the *pup14*, *gtr1*, and *nrt1.7* mutants influenced BrYV infection to different degrees.

## 1. Introduction

Viruses depend on host factors during the infection cycle. There are many studies to identify host factors that are involved in virus infection, including virion particle disassembly [1], viral genome translation [2,3,4,5], viral genome replication [6,7,8,9,10], and viral replication complex composition [11,12,13,14,15,16,17]. After the infection has been established, viruses move between adjacent plant cells via plasmodesmata [18,19,20]. However, there are relatively few studies specifically focused on host factors related to phloem transport. The VPg proteins from potyviruses interact with *Potyvirus* VPg-interacting protein, which acts as an ancillary factor to support potyvirus infection and movement [21]. Pectin methylesterase interacts with tobacco mosaic virus (TMV) movement protein [22], and reduced pectin methylesterase levels in the plant vasculature compromise TMV egress from the infected vasculature, resulting in a significant delay in TMV systemic infection [23]. While the identification of the host factors that facilitate viral systemic movement is critical to understanding viral infection, it is equally important to understand negative regulators of movement. In *A. thaliana,* studies have shown that RTM1, RTM2, and RTM3 can restrict the systemic movement of plant viruses, REM1 and REM2 are located in sieve element to resist tobacco etch virus long-distance movement [24,25,26], and RTM3 can self-interact and interact with RTM1 to restrict long-distance movement of potyviruses [27].

Poleroviruses, belonging to the family *Solemoviridae*, cause serious yield and quality losses worldwide and are mostly transmitted by aphids in a circulative non-propagative but persistent manner [28,29,30]. In plant hosts, poleroviruses have vascular tissue tropism restricted to companion cells, phloem parenchyma cells, and sieve tubes [31]. *Polerovirus* members possess positive-, single-stranded RNA genomes ranging from 5000–6000 nt, with at least one sub-genomic RNA from which the 3′end ORFs are translated. The genomes each contain seven highly overlapping major ORFs; there are two ORFs encoded movement proteins that ensure the transport of viral genomes through plasmodesmata and systemic infection plants [32,33,34]. ORF3a was identified as a small non-AUG-initiated ORF in poleroviruses [35]. Mutations of the turnip yellows virus (TuYV) P3a start codon prevent or increase its expression, preventing viral systemic infections in plants [35]. Recently, DeBlasio demonstrated that P3a formed protein complexes with other potato leafroll virus proteins in plants, suggesting that P3a, MP, and/or the non-assembled form of the RTP work in concert to facilitate potato leafroll virus movement through their interactions with the P3a domain [36].

Brassica yellows virus (BrYV) is a tentative species of the genus *Polerovirus* that is widespread across China, South Korea, and Japan [37,38,39,40]. BrYV can be divided into three genotypes (BrYV-A, B, and C), according to the differences in sequences [41,42]. The full-length genomic clone of BrYV-C has been successfully transferred into *A. thaliana*. The two overexpressing lines obtained, named 111 and 412, exhibit severe symptoms, including dwarfism and purple leaves [43]. Our recent work revealed that proline at the 18th position is an important amino acid site for the function of P3a, which is necessary for BrYV to systemically infect *Nicotiana benthamiana*; P3a and its mutant P3a^P18L^ have the same subcellular localization: both can self-interact in vivo, but P3a^P18L^ seems to form more stable dimer and to have stronger self-interaction than wild type [44]. Despite the advances in structural and functional research on P3a, its protein-interacting targets in host plants are still unknown.

In this study, we used BrYV P3a as bait to screen a plant split-ubiquitin-based membrane yeast two-hybrid (MYTH) cDNA library to explain the functional role of P3a in viral infections. A total of 138 proteins were identified. The results showed that P3a interacted with AtPUP14, AtGTR1, and AtNRT1.7. We further detected the subcellular localizations of these proteins after BrYV infection and co-expression with P3a and their expression levels after BrYV infection. We used *pup14*, *gtr1*, and *nrt1,* the T-DNA insertion mutants, to preliminarily characterize their roles in the BrYV infection process.

## 2. Materials and Methods

### 2.1. Plant Materials and Growth Conditions

*Arabidopsis thaliana* seedlings were vernalized in dark at 4 °C for 2 days, and seedlings were grown at 21 °C under short day (10 h light/14 h dark) and long day (16 h light/8 h dark) conditions for phenotype determination and breeding, respectively. *N. benthamiana* and potted *Solanum tuberosum* plants were grown in a greenhouse with a 16 h light/8 h dark photoperiod at 23–25 °C. We obtained the T-DNA insertion mutants *SALK_004230C* of *AtPUP14* and *SALK_022429C* of *AtNRT1.7* from Prof. Shuhua Yang, and we obtained *SALK_090694.2* of *AtGTR1* from Arashare “https://www.arashare.cn/index/ (accessed on 12 November 2021)”. A genotyping analysis was performed using PCR. The *A. thaliana* line 412 seeds that constitutively expressed the viral genomic RNA of BrYV were used [43].

### 2.2. Plasmid Construction

For the MYTH assay, the coding sequences of *AtGTR1*, *AtPUP14*, *AtNCL*, *AtSTL2P*, *AtNHL3*, *AtRbCS1B*, and *AtNRT1.7* were amplified from *A. thaliana* cDNA, and the coding sequences of *NbNRT1.7*, *NbNCL*, *NbTPT*, *NbMETK2*, *NbKNAT*, *NbBI1*, and *NbLTP1* were amplified from *N. benthamiana* cDNA. They were then independently cloned into the pPR3-N vector at the *Sfi* I site. The plasmids pBT3-STE-P3a and pPR3-N-P3a were described previously [44]. For bimolecular fluorescence complementation (BiFC) assays, *AtPUP14*, *AtGTR1*, *AtNRT1.7*, *AtCB5D*, *AtNCL*, *AtNHL3*, *AtAVA-P1*, *NbTPT*, *NbBI1*, *NbMCU5*, *NbLTP1*, and *NbPVA12* were independently inserted into the pSPYNE-35S vector [45] at the *Bam*HI and *Sal*I sites. The P3a-YN and P3a-YC plasmids were described previously [44]. For subcellular localization assays, *AtPUP14*, *AtGTR1*, and *AtNRT1.7* were independently cloned into the vector pGD-cECFP-6MYC [46]. The plasmid PGDRm-P3a was described previously [44]. For *Agrobacterium*-mediated transient expression assays, *AtPUP14* was inserted into the *Xho*I and *Apa*I sites of the pGD-3Flag vector.

### 2.3. The MYTH Assay

Yeast strain (NMY51) and the plant MYTH cDNA library were obtained from Biogene Biotech (Shanghai, China). For the construction of the plant MYTH cDNA library, the total RNAs of *A. thaliana*, *N. benthamiana*, and *S. tuberosum* were independently extracted using TRIzol reagent (Invitrogen, San Diego, CA, USA). To screen the host proteins to interact with P3a bait against a NubG-fused cDNA library of plants, the plasmid pBT3-STE-P3a was used as bait. An analysis of the autoactivation and toxicity of bait vector (pPR3-STE-P3a) was performed as previously described [44]. The cDNA library was screened in accordance with the DUAL membrane starter kit’s user manual (Dualsystems biotech) and plated on SD/Leu-Trp culture medium (DDO) at 30 °C for 3 days. The positive colonies were patched out onto SD/-Leu/-Trp/-His/-Ade culture medium supplemented with 30 mM 3-AT (QDO/30 mM 3-AT). After 3 days, positive colonies retransferred into SD/-Trp/-Leu liquid medium, cultured for 1 day, and plasmids were isolated using the yeast plasmid extraction kit (Solarbio, Beijing, China). The plasmids were extracted and retransformed into *Escherichia coli* DH5α and then sequenced and analyzed using TAIR “https://www.arabidopsis.org/ (last accessed on 20 November 2022)”, Sol Genomics Network “(https://solgenomics.net (last accessed on 20 November 2022)”, Uniport “https://www.uniprot.org/ (last accessed on 24 November 2022)”, and National Center for Biotechnology Information “https://www.ncbi.nlm.nih.gov/ (last accessed on 24 November 2022)”.

### 2.4. Bioinformatics

To further obtain information on the biological functions and related biological pathways of each P3a primary interacting protein, all the targets were imported into Metascape “http://metascape.org (accessed on 16 April 2022)” and annotated to analyze the functional enrichment and the KEGG pathways [47]. During the analysis, terms with *p* < 0.01, minimum count of 3, and enrichment factor > 1.5 were collected and grouped into clusters. In addition, the Bioinformatics platform “http://www.bioinformatics.com.cn/ (accessed on 16 April 2022)” was used to visualize the GO enrichment analysis.

### 2.5. BiFC Assays and Subcellular Localization

The BiFC assays and subcellular localization were performed as described previously [44]. The infiltrated *N. benthamiana* leaves were observed at 2 days post-inoculation (dpi) using Leica SP8 confocal microscopes. The YFP, CFP, and RFP fluorophores were excited at 488, 405, and 552 nm, respectively.

### 2.6. RNA Extraction and Quantitative Real-Time PCR

Briefly, total RNA was isolated from plants using TRIzol reagent. Total RNAs were treated with recombinant DNase I (TaKaRa, Shiga, Japan) at 37 °C for 1 h and then acted as templates to synthesize first-strand cDNAs with M-MLV reverse transcriptase (Promega, Fitchburg, WI, USA). For the qRT-PCR analysis, gene fragments were amplified with GoTaq^®^ qPCR Master Mix (Promega, Fitchburg, WI, USA). Each reaction system (20 μL) contained 10 μL of 2× real-time PCR mix (containing SYBR Green I), 0.4 μL of PCR forward or reverse primer, and adequate cDNA. The PCR temperature cycling conditions were 95 °C for 2 min, followed by 40 cycles of 95 °C for 15 s, and 60 °C for 1 min. Each treatment was independently repeated three times, and the specific primers are listed in Table S1. The *A. thaliana Actin2* (AT3G18780) gene served as an endogenous control. Student’s *t*-test was employed to determine statistically significant differences compared with the wild type (* *p* < 0.05; ** *p* < 0.01).

### 2.7. Virus Inoculation by Insect Transmission

A virus transmission experiment was performed as described previously [48]. Briefly, *A. thaliana* grown for 4 weeks was used for aphid (*Myzus persicae*) feeding. After feeding on BrYV transgenic *A. thaliana* line 412 for several days, wild-type *A. thaliana* and each T-DNA insert mutant line were inoculated independently using six viruliferous aphids (second–third instar) for 2 days. To determine *AtPUP14*, *AtGTR1*, and *AtNRT1.7* mRNA level changes after BrYV infection, the aphids that fed on healthy *A. thaliana* leaves were used as negative controls.

### 2.8. Agrobacterium-Mediated Transient Expression in N. benthamiana

The *Agrobacterium*-mediated transient expression assays were performed as described previously [49]. One half of *N. benthamiana* leaves were infiltrated with *Agrobacterium tumefaciens* containing BrYV infectious cDNA clone and AtPUP14-3Flag; the other half of *N. benthamiana* leaves were infiltrated with *Agrobacterium tumefaciens* containing BrYV infectious cDNA clone and GFP-3Flag.

### 2.9. Western Blotting Analysis

Total proteins were harvested from plant samples as described previously [49]. Proteins were isolated on 15% SDS polyacrylamide gels and then transferred onto polyvinylidene fluoride membranes (GE Healthcare, Buckinghamshire, UK). The membranes were blotted with a mouse anti-flag antibody (1:5000; Abmart) or rabbit anti-BrYV movement protein antiserum (1:1000) and then incubated with goat anti-mouse HRP antibody (1:3000; Bio-Rad, Hercules, CA, USA) or goat anti-rabbit HRP antibody (1:3000; Sigma-Aldrich, St. Louis, MO, USA), respectively. Finally, the membranes were detected using an enhanced chemiluminescence detection method.

### 2.10. Accession Numbers

Sequence information used in this research is available at TAIR “https://www.arabidopsis.org/ (last accessed on 20 November 2022)”, Sol Genomics Network “(https://solgenomics.net (last accessed on 20 November 2022)”, Uniport “https://www.uniprot.org/ (last accessed on 24 November 2022)”, and National Center for Biotechnology Information “https://www.ncbi.nlm.nih.gov/ (last accessed on 24 November 2022)”, under the following accession numbers: *AtGTR1* (NM_114665), *AtNRT1.7* (NM_105655), *AtPUP14* (NM_101833), *AtNHL3* (NM_120715), *AtRbCS1B* (NM_001344249), *AtNCL* (NM_104200), *AtSTL2P* (NM_126208), *NbPVA12* (Niben101Scf03599g01016.1), *NbNCL* (Niben101Scf07060g03008.1), *NbBI1* (Niben101Scf02705g01020.1), *NbLTP1* (Niben101Scf29144g00011.1), *NbKNAT* (Niben101Scf09454g00008.1), *NbMETK2* (Niben101Scf01861g00002.1), *NbNRT1.7* (Niben101Scf05189g00002.1), and *NbTPT* (Niben101Scf03427g05007.1).

## 3. Results

### 3.1. Identifying BrYV P3a-Interacting Host Proteins by Yeast Library Screening

Because BrYV-P3a is a predicted transmembrane protein, we constructed a MYTH cDNA library from *A. thaliana*, *N. benthamiana*, and *S. tuberosum* to identify host proteins that potentially interact with P3a. The results indicated that the library was of high quality and could be used for screening of P3a-interaction proteins. Our previous study indicated the bait is functional in the DUAL membrane system, and there was no auto-activation of pBTS-STE-P3a [44]. To screen host proteins that interplay with the P3a bait, the pBTS-STE-P3a plasmid and cDNA library plasmid were co-transformed into NMY51. After screening, 630 yeast clones were collected from the SD/-Leu/-Trp/-His/-Ade agar plates supplemented with 30 mM 3-AT (QDO/30 mM 3-AT). In total, 138 genes were obtained by colony PCR amplification and sequentially sequenced. Among them, 53 genes were screened in *A. thaliana*, 42 genes in *N. benthamiana*, and 43 genes in *S. tuberosum*. We used Uniport to annotate functions of proteins of the acquired clones, as shown in Table S2.

**Figure 1:**
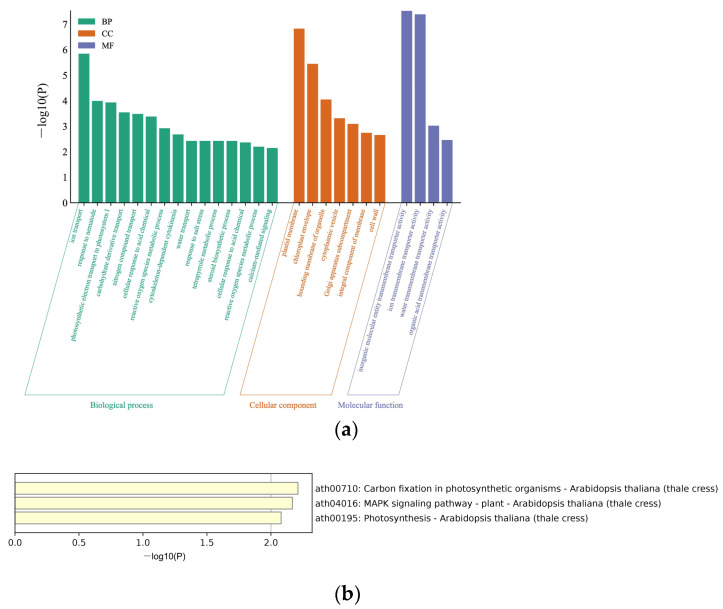
GO term and KEGG enrichment analyses. (**a**) GO functions of screened host factors. The horizontal axis represents the specific terms of biological processes, cellular components, and molecular functions. The vertical axis represents the *p*-value. The bars represent the GO functions enriched with target proteins; the horizontal coordinates are sorted according to the *p*-value; (**b**) KEGG pathway enrichment analysis. The horizontal axis represents the *p*-values of pathways, and the vertical axis represents the pathways.

To further explore the enrichment functions of the 138 proteins, the Metascape database was used to analyze the Gene Ontology (GO) annotation and the Kyoto Encyclopedia of Genes and Genomes (KEGG) pathway annotation and enrichment analysis. The GO annotation grouped all the screened proteins into three different GO categories, with 15 terms in biological processes, seven terms in cellular components, and four terms in molecular functions. The biological processes, such as ion transport, response to nematode, and photosynthetic electron transport in photosystem (PS) I, were enriched. The enriched cellular components were plasma membrane, chloroplast membrane, and bounding membrane of organelle. Furthermore, the enriched molecular function categories were dominated by inorganic molecular entity transmembrane transport activity, ion transmembrane transport activity, and water transmembrane transport activity (Figure 1a). Additionally, KEGG was used to analyze the pathway enrichment of the 138 proteins, and 3 KEGG pathways for the acquired proteins were identified: carbon fixation in photosynthetic, MAPK signaling pathway, and photosynthesis (Figure 1b).

### 3.2. Validation of P3a-Interacting Proteins

**Figure 2:**
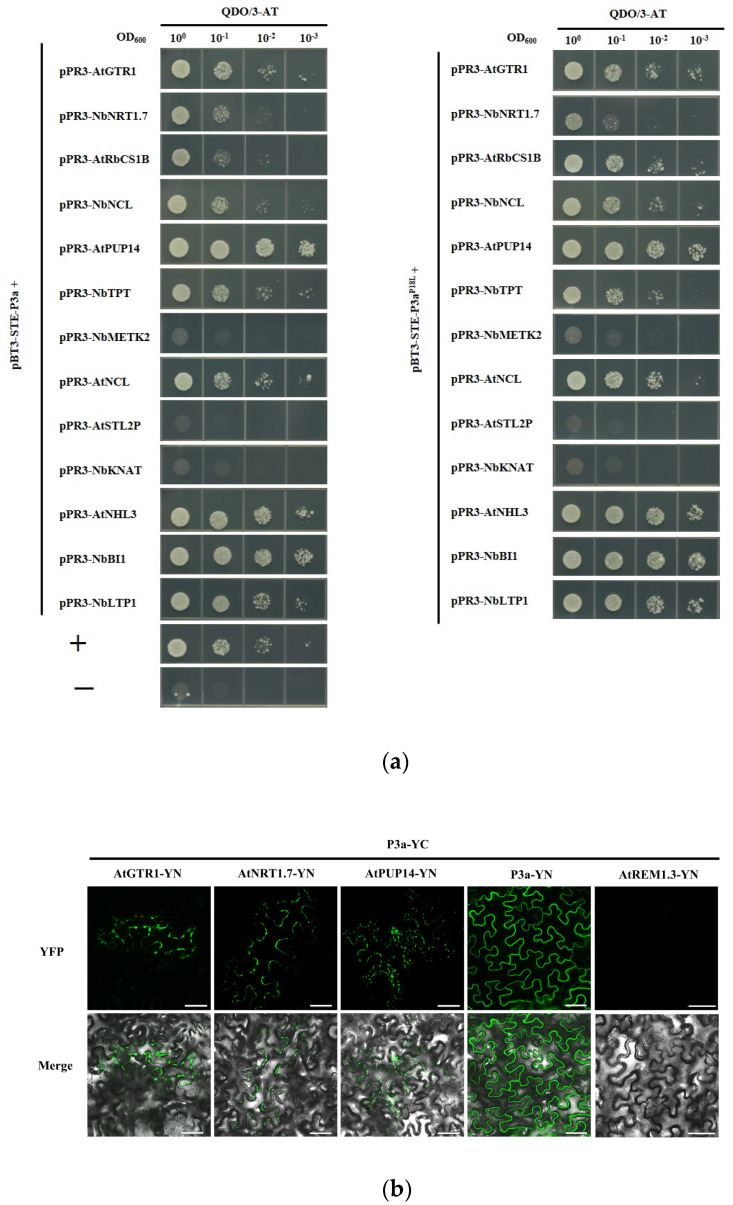
Verification of the interactions between P3a (P3a^P18L^) and screened host factors. (**a**) Analysis of the interactions between P3a(P3a^P18L^) and 13 screened host proteins in the MYTH system. The interactions were indicated by yeast growth on QDO/30 mM 3-AT. P3a self-interaction and pPR3-N + pBT3-STE-P3a served as the positive and negative controls, respectively; (**b**) BiFC assay of interactions between P3a and the three candidate interacting proteins. Self-interaction of P3a served as a positive control. AtREM1.3-YN with P3a-YC served as a negative control. Scale bars = 50 μM.

Based on the screened data, 13 clones involved in different pathways were selected for further research. The full-length cDNAs of the 13 genes were individually amplified and constructed into the prey vector pPR3-N to generate pPR3-N fusion clones. They were then each co-transformed with pBT3-STE-P3a into the yeast strain NMY51 and cultured on DDO for 3 days at 30 °C. The yeast cell co-transformations of pBT-STE-P3a with all the prey vectors produced clones on the DDO culture medium. These clones were cultured and plated in 10-fold serial dilutions and grown on QDO/30 mM 3-AT culture medium. After 3 days, the yeast cell co-transformations of pBT3-STE-P3a with pPR3-N-AtGTR1, pPR3-N-NbNRT1.7, pPR3-N-AtRbCS1B, pPR3-N-AtPUP14, pPR3-N-NbNCL, pPR3-N-NbTPT, pPR3-N-AtNCL, pPR3-N-AtNHL3, pPR3-N-NbBI1, and pPR3-N-NbLTP1, as well as the positive control, produced colonies on the QDO/30 mM 3-AT culture medium. These were selected as the candidate partners of P3a. However, pBT3-STE-P3a with pPR3-N-AtSTL2P, pPR3-N-NbMETK2, and pPR3-N-NbKNAT, as well as the negative control, showed no growth on QDO/30 mM 3-AT culture medium. In addition, we determined whether these P3a-interacting candidate partners also interacted with P3a^P18L^. The proteins that interacted with P3a also interacted with P3a^P18L^, whereas the proteins that did not interact with P3a did not interact with P3a^P18L^, which suggested that these 13 proteins may not be involved in the systemic transport function of P3a (Figure 2a).

To investigate whether the proteins that interacted with P3a in yeast also interacted in plant cells, we performed a BiFC assay. The C-terminal domain of yellow fluorescent protein (YFP)-fused P3a was transiently expressed with each of 12 other host proteins that were fused independently with the N-terminal domain of YFP in *N. benthamiana* leaves. They were then visualized using confocal microscopy at 2 dpi. Fluorescent signals from the interaction between P3a and AtPUP14, AtGTR1, and AtNRT1.7 were observed at the cell periphery (Figure 2b), whereas BiFC fluorescence was not observed in any of the nine combinations.

### 3.3. P3a and BrYV hijack P3a-Interacting Proteins from the Plasma Membrane to Cytosol

**Figure 3:**
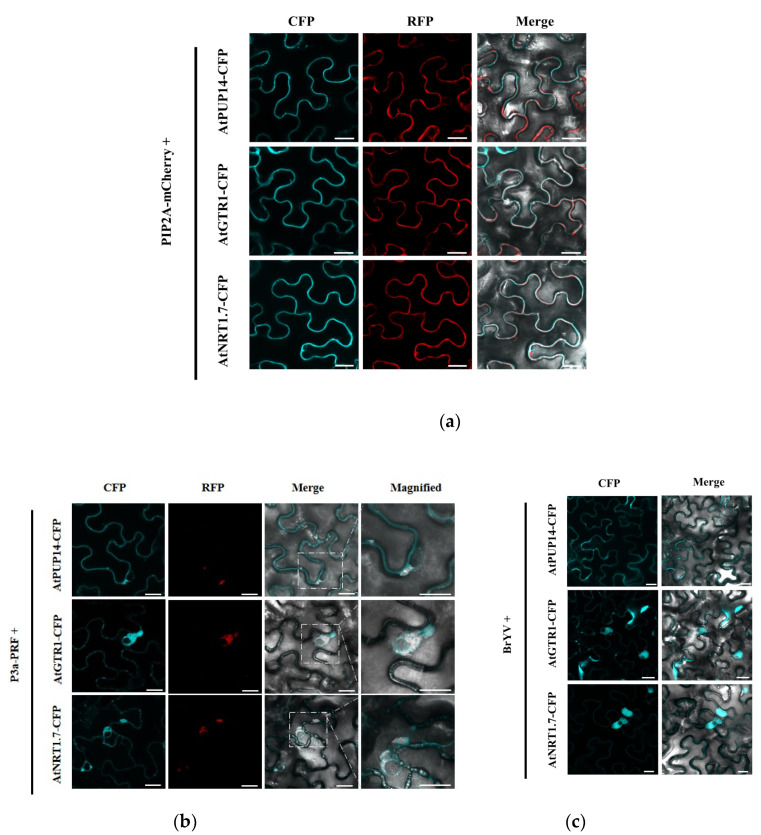
Subcellular localization of P3a interacting proteins during P3a expression and BrYV infection. (**a**) Co-localization of P3a and PIP2A-mCherry. PIP2A acts as a PM marker; (**b**) Co-localization of P3a and its interacting proteins. AtPUP14-CFP, AtGTR1-CFP, and AtNRT1.7-CFP were co-expressed with P3a-RFP in *N. benthamiana* leaves; (**c**) Subcellular localizations of AtPUP14-CFP, AtGTR1-CFP, and AtNRT1.7-CFP during BrYV infection; confocal analyses were conducted at 2 dpi. Scale bars = 20 μM.

Plasma membrane intrinsic protein (PIP2) is a well-known plasma membrane (PM) localization protein [50]. To confirm its localization, AtPUP14-CFP, AtGTR1-CFP, and AtNRT1.7-CFP were independently transiently co-expressed with PIP2A-mCherry in *N. benthamiana* leaves by agroinfiltration. As expected, AtPUP14-CFP, AtGTR1-CFP, and AtNRT1.7-CFP co-localized with PIP2A-mCherry in the PM (Figure 3a). Cytological assays were performed to determine whether AtPUP14, AtGTR1, and AtNRT1.7 colocalized with P3a. In *N. benthamiana* leaves co-expressing P3a-RFP and the P3a interacting proteins, P3a altered the PM location of the interacting proteins. AtPUP14 co-localized with P3a in the cytosol and formed aggregates, whereas AtGTR1-CFP and AtNRT1.7-CFP did not overlap with P3a-RFP. They were adjacent to P3a-RFP and accumulated as irregularly shaped perinuclear aggregates (Figure 3b). During the infection of *N. benthamiana* by BrYV, AtGTR1 and AtNRT1.7 moved from the PM to the cytosol, which was similar to the co-localization with P3a, but there was no obvious change in the localization of AtPUP14 (Figure 3c).

### 3.4. Expression Levels of AtPUP14, AtGTR1, and AtNRT1.7 in Response to BrYV

**Figure 4:**
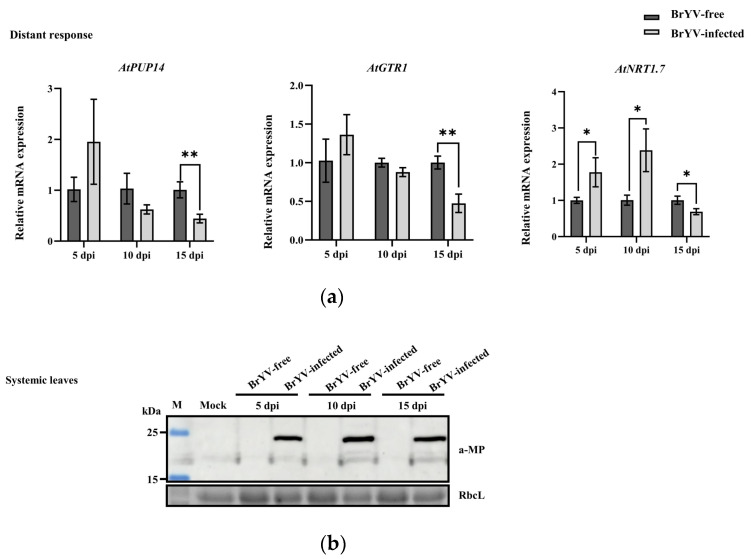
Relative mRNA levels of the *AtPUP14*, *AtGTR1*, and *AtNRT1.7* genes in response to BrYV infection. (**a**) Relative mRNA levels of the *AtPUP14*, *AtGTR1*, and *AtNRT1.7* genes in response to BrYV infection at 5, 10, and 15 dpi in systemic leaves. The non-viruliferous aphids were used as a negative control, and *AtActin2* was used as an internal control. Student’s t-test was employed to determine statistically significant differences (* *p* < 0.05; ** *p* < 0.01); (**b**) Detection of BrYV-MP accumulation in *A. thaliana* systemic leaves inoculated with viruliferous aphids or non-viruliferous aphids.

We measured the transcript levels of *AtPUP14*, *AtGTR1*, and *AtNRT1.7* in response to BrYV infection. For *AtPUP14*, the expression level in systemic leaves was upregulated at 5 dpi with BrYV, downregulated at 10 dpi, and finally showed significant down-regulation at 15 dpi. For *AtGTR1*, the expression level in systemic leaves was significantly downregulated at 15 dpi with BrYV, whereas there were no significant changes in expression levels at 5 and 10 dpi to BrYV infection. For *AtNRT1.7*, the expression level was significantly upregulated at 5 and 10 dpi, followed by a significant downregulation at 15 dpi (Figure 4a). We then detected BrYV-MP accumulation in systemic leaves of *A. thaliana* inoculated with viruliferous aphids or non-viruliferous aphids. MP was only detected in the systemic leaves inoculated with viruliferous aphids (Figure 4b). Thus, *AtPUP14*, *AtGTR1*, and *AtNRT1.7* responded to BrYV infection. Additionally, the expression levels of these genes were different in response to BrYV infection, and the same gene had different expression levels after different durations of BrYV infection.

### 3.5. The Effects of A. thaliana T-DNA Insertion Mutants pup14, gtr1, and nrt1.7 on BrYV Infection

**Figure 5:**
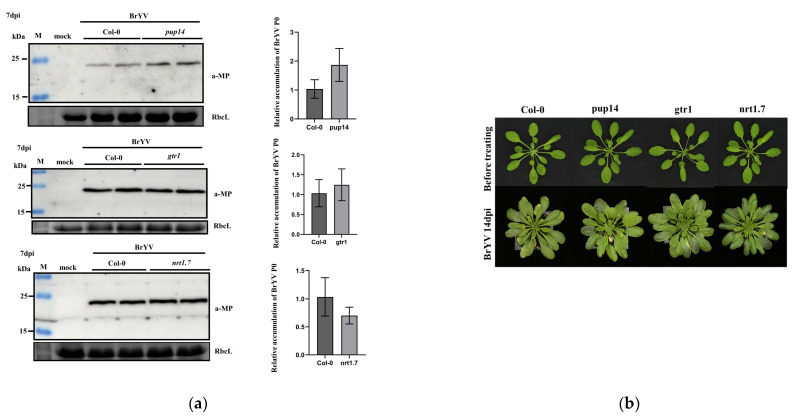
Effects of T-DNA insertion mutants of P3a-interacting proteins on BrYV accumulation. (**a**) Western blotting showing the accumulation of BrYV-MP in systemically infected leaves at 7 dpi. RbcL served as a loading control. qRT-PCR analyses of viral RNA accumulation; *AtActin2* was used as an internal control; (**b**) Development and symptom induction of Col-0, *pup14*, *gtr1*, and *nrt1.7* under normal conditions or BrYV infection at 14 dpi.

We next investigated the functions of three P3a interacting proteins during BrYV infection. First, the DNA of seedlings was extracted to test these mutants. The wild-type *A. thaliana* was only able to amplify bands corresponding to primers LP and RP, whereas T-DNA insertion mutants were only able to amplify bands corresponding to primers LB1.3 and RP, which indicated that these mutants were homozygous. Both Col-0 and T-DNA insertion mutants were inoculated with viruliferous aphids for 2 days. At 7 dpi, *A. thaliana* systemic leaves were collected, and total protein and RNA were extracted for testing. Western blotting and qRT-PCR analyses showed that BrYV-MP protein and BrYV RNA levels slightly increased in mutant *pup14* compared with Col-0. However, there were no significant changes in MP accumulation in *gtr1* and *nrt1.7* compared with Col-0 (Figure 5a). Before the BrYV inoculation, no obvious phenotypic differences were observed between the wild type and T-DNA insertion mutants. After BrYV treatment, T-DNA insertion mutants did not develop more symptoms at 14 dpi compared with Col-0 plants (Figure 5b). To further verify the function of AtPUP14 in BrYV infection, we used *Agrobacterium*-mediated transient expression assays, co-infiltrated *Agrobacterium* containing BrYV and AtPUP14-3Flag into *N. benthamiana* leaves, and used BrYV and GFP-3Flag as a control. The results showed overexpression of AtPUP14 decreased the BrYV-MP accumulation level in *N. benthamiana* leaves (Figure S1).

## 4. Discussion

In the genus *Polerovirus*, BrYV is a tentative newly identified species, and it is closely related to TuYV [41,51]. The P3a protein encoded by ORF3a of the genus *Polerovirus*, is required for long-distance movement in plants [35,44]; however, the mechanism is still not clear. This is mainly due to a lack of knowledge regarding the host factors with which P3a interacts during the phloem trafficking process. Because P3a is a predicted transmembrane protein, 138 host proteins interacting with P3a have been screened using the MYTH method. These 138 proteins belong to three different species, respectively, and some of these proteins can be screened in more than one species, suggesting that amino acids that interact with P3a in yeast are conserved across these species. Some proteins can only be screened in one species, which may be due to the fact that frequencies of different proteins in the yeast cDNA library are different, causing the low frequency proteins to be missed. Otherwise, the interactions between viral proteins and plant proteins may be different in different species, causing the same virus infecting different hosts to have various molecular partners and events. The carbon fixation in photosynthetic, the photosynthesis pathway, and the MAPK signaling pathway enriched with P3a protein interactions may play essential roles in BrYV infection.

Photosynthesis is the most important metabolic process of plants, providing the necessary materials and energy for all life activities. It consists of photosynthetic phosphorylation, carbon assimilation, and photosynthetic product synthesis. PSI and PSII are important sites for biological photovoltaic energy conversion, which involve key steps, such as hydrolysis and primary charge separation, and they are important sites for determining photosynthetic efficiency [52]. The PSI core of higher plants consists of 14 protein subunits. In addition to the two core proteins PsaA and PsaB, they all contain three hydrophilic subunits and five peripheral proteins. PsaL, which interacts with P3a, is among the peripheral proteins. P3a also interacts with some proteins of PSII complexes. PsbR and PsbS are the proteins we screened that interact with P3a. PSbR plays an important role in stabilizing PSII complex [53]. As for PsbS, experiments have also shown that PsbS can be used to improve water use efficiency and enhance ROS homeostasis in plants [54,55]. Both proteins play an active role in plant growth, and P3a may promote viral infection by interfering with the stability of these proteins or affecting their interactions with other proteins. Ribulose 1,5-bisphosphate carboxylase/oxygenase (Rubisco) plays an important role in plant photosynthesis, and the enzyme plays a key role in the carbon dioxide fixation. The contents of chloroplasts and their internal morphology are generally affected after a viral infection. When tomato mosaic virus infects *N. benthamiana*, virus movement protein interacts with the host rubisco small subunit (RbCS) protein, in addition NbRbCS promotes virus movement and plant antiviral defenses [56]. In Arabidopsis, there are four members, RBCS1A, RBCS1B, RBCS2B, and RBCS3B. Here, we determined that RBCS1B and RBCS3B may interact with P3a; therefore, we speculate that P3a may facilitate virus infection by affecting photosynthesis.

Another class of proteins we screened that interact with P3a are involved in the MAPK signaling pathway, whereas previous studies have shown that plant viruses can both activate and inhibit MAPK; for example, βC1 selectively interferes with the MAPK signaling pathway, thereby resisting the viral defense response and facilitating viral infection [57]. P3a may influence BrYV infection by disturbing the MAKP signaling pathway.

In addition, we cloned genes of interest for interaction verification and determined that some proteins interact with P3a and also with P3a^P18L^ in yeast, indicating that these genes may not be the key factors in disrupting the system movement function of the P3a^P18L^. However, MYTH screens always yield a varying number of false-positive candidates; therefore, all candidates need to be confirmed by biologically relevant cellular techniques. The BiFC assay is a powerful technology for the further verification of MYTH results. Ultimately, we found three *A. thaliana* phloem-localized proteins, AtPUP14, AtGTR1, and AtNRT1.7, that could be detected as interacting proteins with P3a using both methods. AtPUP14 regulates the spatial and temporal patterns of cytokinin by importing extracellular cytokinin into the cells [58]. AtGTR1 and AtNRT1.7 are both members of the nitrate/peptide transporter family, and the main role of AtGTR1 is to regulate the level of GLSs in seedlings [59,60]. GLSs are a group of secondary metabolites involved in defenses against herbivores. In addition, AtNRT1.7 shows a 3% uptake activity compared with GTR1′s glucosinolate transport activity; the main function of AtNRT1.7 is the transport of nitrate in phloem [59,61]. Host genes’ expression are influenced by virus infection [62,63], resulting in the upregulation of susceptibility genes and the activation or downregulation of antiviral defense responses. The expression levels of these three P3a-interacting proteins were up-regulated during the early stage of BrYV infection and down-regulated during the late stage of BrYV infection. The *AtPUP14* expression showed a down-regulation trend during late BrYV infection stages, which was consistent with the transcriptome results of transgenic Arabidopsis lines that constantly express the genomic RNA of BrYV [43]. In addition, it has been shown that in *Brassica napus, BN14638* gene expression was up-regulated only in resistant cultivar after *Sclerotinia sclerotiorum* infection; as a homolog of *AtPUP14*, it may play a role in the immune response [64]. Expression of P3a protein resulted in altered membrane localization of all three proteins. We then examined the effect of these three proteins on virus infection using T-DNA insertion mutants. AtPUP14 had an effect on BrYV infection; however, AtGTR1 and AtNRT1.7 had no significant effect on BrYV; it could be that there are genes with redundant functions in *A. thaliana*. Here, there was only one line of T-DNA insertion mutants of each protein to study their effects on BrYV infection. In the future, we will use other knockout mutants or generate overexpression plants to refine our investigation.

## 5. Conclusions

In this study, we systemically investigated BrYV-P3a interacting proteins in plants. Bioinformatics analysis revealed that the genes from carbon fixation in photosynthetic, photosynthesis pathways, and MAPK signaling were affected. Furthermore, AtPUP14, AtGTR1, and AtNRT1.7 were verified to interact with P3a in vivo. We demonstrated that P3a and these three interacting proteins mainly co-localized in the cytoplasm. Expression levels of *AtPUP14*, *AtGTR1*, and *AtNRT1.7* were significantly reduced in response to BrYV during the late stages of viral infection, and the *pup14*, *gtr1*, and *nrt1.7* mutants influenced BrYV infection to different degrees. Overall, the screening and identification of P3a-interacting host factors enhanced the understanding of the molecular mechanisms of BrYV infection.

## Data Availability

The data presented in this study are available within the article.

## Supplementary Materials

The following supporting information can be downloaded at: https://www.mdpi.com/article/10.3390/biology12020202/s1, Table S1: Sequences of primers used in this study. Table S2: Sequencing analyses results of the candidate genes that encode host proteins potentially interacting with P3a. Figure S1: Overexpression of AtPUP14 decreased local BrYV accumulation level in inoculated leaves.
