# Supplementary material for: Identification and Functional Analyses of Host Proteins Interacting with the P3a Protein of Brassica Yellows Virus
Source: Biology (Basel). 2023 Jan 28;12(2):202. doi: 10.3390/biology12020202 (PMC9952887; doi:10.3390/biology12020202)
Supplement: Supplementary file 1 [file biology-12-00202-s001.zip › biology-2097572-supplementary.pdf]

Table S1. Sequences of primers

Test name	Primer	Sequence (5'-3')
MYTH		
	PPR3-N-AtGTR1-F	ATTAACAAGGCCATTACGGCCATGAAGAGCAGAGTCATTCT TAACC
	PPR3-N-AtGTR1-R	AACTGATTGGCCGAGGCGGCCTCAGACAGAGTTCTTGTCTT GTAGC
	PPR3-N-AtNRT1.7-F	ATTAACAAGGCCATTACGGCCATGGTTTTGGAGGATAGAAA GGACG
	PPR3-N-AtNRT1.7-R	AACTGATTGGCCGAGGCGGCCTCATTTCATCGATTTCTTCGA AGTC
	PPR3-N-AtPUP14-F	ATTAACAAGGCCATTACGGCCATGGCTCAGAATCAACAACC TATTT
	PPR3-N-AtPUP14-R	AACTGATTGGCCGAGGCGGCCCTAATAAGCCATACGATTGT CTTTG
	PPR3-N-AtNCL-F	ATTAACAAGGCCATTACGGCCATGAGATTCAGATCTCTCAT CTCTC
	PPR3-N-AtNCL-R	AACTGATTGGCCGAGGCGGCCCTACGACCAGCCAAACCAG TAATCA
	PPR3-N-AtSTL2P-F	ATTAACAAGGCCATTACGGCCATGGCGAATCAGAGTACAG AGACGA
	PPR3-N-AtSTL2P-R	AACTGATTGGCCGAGGCGGCCCTAAGGTATGATACCCTTTG CCTTC
	PPR3-N-AtNHL3-F	ATTAACAAGGCCATTACGGCCATGGCGGACTTAAACGGTGC GTATT
	PPR3-N-AtNHL3-R	AACTGATTGGCCGAGGCGGCCTCAAAAGTCAACGTCACAC TTGGTC
	PPR3-N-AtRbCS1B-F	ATTAACAAGGCCATTACGGCCATGGCTTCCTCTATGCTCTCC TCTG
	PPR3-N-AtRbCS1B-R	AACTGATTGGCCGAGGCGGCCTTAAGCATCAGTGAAGCTTG GGGGC
	PPR3-N-NbNRT1.7-F	ATTAACAAGGCCATTACGGCCATGGAGAACAAGAAAAACC AGATGA
	PPR3-N-NbNRT1.7-R	AACTGATTGGCCGAGGCGGCCTTAATACTTCATGTCTTGAA GCTCA
	PPR3-N-NbNCL-F	ATTAACAAGGCCATTACGGCCATGTTACCCAAAGCTTATCTT TCTA
	PPR3-N-NbNCL-R	AACTGATTGGCCGAGGCGGCCCTATGACCAACCAAATACG TAGTCA
	PPR3-N-NbTPT-F	ATTAACAAGGCCATTACGGCCATGGAGTCTCGCGTTTTGAC TGGCG
	PPR3-N-NbTPT-R	AACTGATT

		GGCCGAGGCGGCCTCAGGCAGCTTTCTTTTGCCTTTTC
	PPR3-N-NbMETK2-F	ATTAACAAGGCCATTACGGCCATGGAAACTTTCCTATTTACCTCCG
	PPR3-N-NbMETK2-R	AACTGATTGGCCGAGGCGGCCTTAGTCTTGGGGCTTTTCCCTCTTG
	PPR3-N-NbKNAT1-F	ATTAACAAGGCCATTACGGCCATGGCGTTTCAGGACCATTTTTCTC
	PPR3-N-NbKNAT1-R	AACTGATTGGCCGAGGCGGCCTCACTGCTTGATTTCACCTGCACTC
	PPR3-N-NbBI1-F	ATTAACAAGGCCATTACGGCCATGGAAGGTTTTACCTCGTTCTTCA
	PPR3-N-NbBI1-R	AACTGATTGGCCGAGGCGGCCCTAGTCTCTCCTCTTCTTCTTCGTC
	PPR3-N-NbLTP1-F	ATTAACAAGGCCATTACGGCCATGGCTAAAGCAGCATTGTTGGTGG
	PPR3-N-NbLTP1-R	AACTGATTGGCCGAGGCGGCCTTACTGCACCGTGGAGCAGTCAATG
BiFC		
	NE-AtGTR1-F	CGCGCCACTAGTGGAATGAAGAGCAGAGTCATTCT
	NE-AtGTR1-R	ACCCTCGAGGTCGACGACAGAGTTCTTGTCTTGTA
	NE-AtNRT1.7-F	CGCGCCACTAGTGGAATGGTTTTGGAGGATAGAAA
	NE-AtNRT1.7-R	ACCCTCGAGGTCGACTTTCATCGATTTCTTCGAAG
	NE-AtPUP14-F	CGCGCCACTAGTGGAATGGCTCAGAATCAACAACC
	NE-AtPUP14-R	ACCCTCGAGGTCGACATAAGCCATACGATTGTCTT
	NE-AtCB5D-F	CGCGCCACTAGTGGAATGGGCGGAGACGGAAAAGT
	NE-AtCB5D-R	ACCCTCGAGGTCGACAGAAGAAGGAGCCTTGGTCT
	NE-AtNCL-F	CGCGCCACTAGTGGAATGAGATTCAGATCTCTCAT
	NE-AtNCL-R	ACCCTCGAGGTCGACCGACCAGCCAAACCAGTAAT
	NE-AtNHL3-F	CGCGCCACTAGTGGAATGGCGGACTTAAACGGTGC
	NE-AtNHL3-R	ACCCTCGAGGTCGACAAAGTCAACGTCACACTTGG
	NE-AtAVA-P1-F	CGCGCCACTAGTGGAATGTCTACGTTCAGCGGCGA
	NE-AtAVA-P1-R	ACCCTCGAGGTCGACTTCAGCTCTAGACTGGCCAG
	NE-NbTPT-F	CGCGCCACTAGTGGAATGGAGTCTCGCGTTTTGAC
	NE-NbTPT-R	ACCCTCGAGGTCGACGGCAGCTTTCTTTTGCCTTT
	NE-NbBI1-F	CGCGCCACTAGTGGAATGGAAGGTTTTACCTCGTT
	NE-NbBI1-R	ACCCTCGAGGTCGACGTCTCTCCTCTTCTTCTTCG
	NE-NbMCU5-F	CGCGCCACTAGTGGAATGTGGAGAACTTCGTTTAA
	NE-NbMCU5-R	ACCCTCGAGGTCGACTGTGCAAGCTCATCAATTTT
	NE-NbLTP1-F	CGCGCCACTAGTGGAATGGCTAAAGCAGCATTGTT
	NE-NbLTP1-R	ACCCTCGAGGTCGACCTGCACCGTGGAGCAGTCAA
	NE-NbPVA12-F	CGCGCCACTAGTGGAATGTCGTCGACATTCAGCGG
	NE-NbPVA12-R	ACCCTCGAGGTCGACCTCTGCTCTGGACTGCCCAG
Locolization		

	PGD-AtPUP14-CFP-F	AGATCTCGAGCTCAAATGGCTCAGAATCAACAA
	PGD-AtPUP14-CFP-R	AGAATTCGAAGCTTGATAAGCCATACGATTGTC
	PGD-AtGTR1-CFP-F	AGATCTCGAGCTCAAATGAAGAGCAGAGTCATT
	PGD-AtGTR1-CFP-R	AGAATTCGAAGCTTGGACAGAGTTCTTGTCTTG
	PGD-AtNRT1.7-CFP-F	AGATCTCGAGCTCAAATGGTTTTGGAGGATAGA
	PGD-AtNRT1.7-CFP-R	AGAATTCGAAGCTTGTTTCATCGATTTCTTCGA
Detect T-DNA insertion mutants		
	SALK_004230C-LP	ACTTGACCCTAAAGCAGGCTC
	SALK_004230C-RP	TCAACCAATCAAAAACCAAGC
	SALK_090694.2-LP	TCTGATGGCGAGAAGCTTATG
	SALK_090694.2-RP	GAACCGAACCGGATTCTTTAC
	SALK_022429C-LP	GGAAGAGATACGCAAGTGGTC
	SALK_022429C-RP	ATAGGTGACAGTGCCGTCATC
	LB1.3	ATTTTGCCGATTTCGGAAC
qRT-PCR		
	qRT-AtPUP14-F	CGAGCGTGTTGTCGAATGTG
	qRT-AtPUP14-R	GGCAGTAACCAAGGCAACAC
	qRT-AtGTR1-F	TTGGCTGGTATTGCTCGTGT
	qRT-AtGTR1-R	AACTTCTCCTCGGGGGTCAT
	qRT-AtNRT1.7-F	GCTGGTTCACTCTCCGTCAT
	qRT-AtNRT1.7-R	TGCCCGCAACGATCATAGAA
	qRT-AtActin2-F	GCACCCTGTTCTTCTTACCG
	qRT-AtActin2-R	AACCCTCGTAGATTGGCACA
	qP-BrC-281F	CCAGAGCTATGTGCCTGGTT
	qP-BrC-410R	TGCTCGCAAGAGATCGTTGT
RT	HC511-18TR	GGATATCTGCAGGATCCAAGCTTTTTTTTTTTTTTTTTT
	PocoCPR	CGTCTACCTATTTSGGRTTN

Table S2. Sequencing analyses results of the candidate genes that encode host proteins potentially interacting with P3a.

Protein Name	Accession Number	Specie and Accession Number of Arabidopsis thaliana Homologues	Function Description in Arabidopsis thaliana	Clone Number
Amino acid transporter AVT6A	NM_113938	*Arabidopsis thaliana*	Involved in exporting amino acids	1
Sodium/calcium exchanger NCL	NM_104200	*Arabidopsis thaliana*	Participates in the maintenance of calcium homeostasis. Involved in salt stress response	3
Major facilitator superfamily protein	NM_119661	*Arabidopsis thaliana*	Function in the uptake of sugars	1
Squamosa promoter-binding-like protein 5	NM_112390	*Arabidopsis thaliana*	Involved in regulation of flowering and vegetative phase change	1
Cytochrome b5 isoform D	NM_124258	*Arabidopsis thaliana*	Membrane bound hemoprotein functions as an electron carrier for several membrane bound oxygenases, including fatty acid desaturases	3
Photosystem II subunit X	NM_126649	*Arabidopsis thaliana*	Involved in the integrity of PSII	1
NDR1/HIN1-like protein 3	NM_120715	*Arabidopsis thaliana*	Confers resistance to *Pseudomonas syringae* pv. tomato DC3000	6
Nodulin MtN21/EamA-like transporter family protein	NM_113723	*Arabidopsis thaliana*	Involved in response to auxin in adventitious root formation	1
Protein NRT1/ PTR FAMILY 2.10	NM_114665	*Arabidopsis thaliana*	Involved in the distribution of glucosinolates within the leaf, including import into the glucosinolate-rich S-cells located adjacent to the phloem.	1
Synaptobrevin-related protein 1	NM_001036392	*Arabidopsis thaliana*	Involved in mediating the post-Golgi trafficking of auxin transporters to the plasma membrane from the TGN subdomains.	1
ER membrane protein	NM_101730	*Arabidopsis thaliana*	Plays important roles in response to ABI1.	1
Protein At14a	NM_113748	*Arabidopsis thaliana*	May serve as an anchor point for Agrobacteria	1
Sugar transporter ERD6	NM_100765	*Arabidopsis thaliana*	Involved in sucrose transporter	1
Protein S-acyltransferase 24	NM_122042	*Arabidopsis thaliana*	Involved in cell growth regulation	1
Outer envelope membrane protein	NM_116181	*Arabidopsis thaliana*	Tail-anchored membrane protein which possesses a single C-terminal transmembrane domain targeting post-translationally to plastids	1
Cytochrome b561 and DOMON domain-containing protein	NM_122964	*Arabidopsis thaliana*	May act as a catecholamine-responsive trans-membrane electron transporter	4

V-type proton ATPase subunit c5	NM_119638	*Arabidopsis thaliana*	Proton-conducting pore forming subunit of the membrane integral V0 complex of vacuolar ATPase.	1
TCP family transcription factor	NM_120916	*Arabidopsis thaliana*	Acts as a repressor of CCA1 by binding to its promoter	1
Plant natriuretic peptide A	NM_179648	*Arabidopsis thaliana*	Plays a systemic role in water and solute homeostasis	1
Calcium-transporting ATPase 4	NM_129719	*Arabidopsis thaliana*	This magnesium-dependent enzyme catalyzes the hydrolysis of ATP coupled with the translocation of calcium from the cytosol into small vacuoles	1
FATTY ACID EXPORT 6	NM_112943	*Arabidopsis thaliana*	It May be involved in free fatty acids export	1
Photosystem I reaction center subunit XI	NM_117349	*Arabidopsis thaliana*	Encodes subunit L of photosystem I reaction center	1
Probable inactive poly [ADP-ribose] polymerase SRO5	NM_125646	*Arabidopsis thaliana*	Its presence suggests a role for the protein in ADP ribosylation	1
Sodium-dependent phosphate transport protein 1	NM_128519	*Arabidopsis thaliana*	May act as an ascorbate transporter at the thylakoid membrane	1
Glutaryl-CoA dehydrogenase	NM_101625	*Arabidopsis thaliana*	Late embryogenesis abundant hydroxyproline-rich glycoprotein family	1
Callose synthase 3	NM_121303	*Arabidopsis thaliana*	Involved in callose synthesis at the forming cell plate during cytokinesis	2
Cytochrome B5 isoform C	NM_130230	*Arabidopsis thaliana*	Function as an electron carrier for several membrane bound oxygenases, including fatty acid desaturases	1
BAG family molecular chaperone regulator 4	NM_115037	*Arabidopsis thaliana*	Co-chaperone that regulates diverse cellular pathways	1
UDP-rhamnose/UDP-galactose transporter 1	NM_001334739	*Arabidopsis thaliana*	Nucleotide-sugar transporter that transports UDP-rhamnose or UDP-galactose and UMP in a strict counter-exchange mod	1
ER lumen protein retaining receptor family protein	NM_120039	*Arabidopsis thaliana*	ER retention sequence binding	1
Putative type 1 membrane protein	NM_113321	*Arabidopsis thaliana*	Plays an important role in the maintenance of intracellular ion homeostasis in response to salt and drought stress	1
HR-like lesion-inducing protein-like protein	NM_001344484	*Arabidopsis thaliana*	Associated with the hypersensitive response pathway of defense against plant pathogens	1
Purine permease 14	NM_101833	*Arabidopsis thaliana*	Purine permease is implicated in ATP-dependent cytokinin translocation that controls the	1

			spatiotemporal landscape of cytokinin signaling.	
Pathogen and circadian controlled 1	NM_113121	*Arabidopsis thaliana*	Regulator of the flowering time in response to stress	1
Serine/threonine-protein kinase D6PK	NM_124973	*Arabidopsis thaliana*	Protein kinase that regulates the auxin transport activity of PIN auxin efflux facilitators by direct phosphorylation	1
Endoplasmic reticulum transmembrane protein	NM_103740	*Arabidopsis thaliana*	It May plays a role the in anterograde transport of membrane proteins from the endoplasmic reticulum to the Golgi	1
Arabinogalactan protein 15	NM_121212	*Arabidopsis thaliana*	Proteoglycan seems to be implicated in diverse developmental roles	1
Cellulose synthase-like protein E1	NM_104462	*Arabidopsis thaliana*	Thought to be a Golgi-localized beta-glycan synthase that polymerizes the backbones of noncellulosic polysaccharides of plant cell wall	1
Integral membrane HRF1 family protein	NM_115812	*Arabidopsis thaliana*	Involved in the endoplasmic reticulum to Golgi vesicle-mediated transport	1
DELLA protein RGA	NM_126218	*Arabidopsis thaliana*	Involved in the regulation of seed dormancy and germination	1
Outer envelope pore protein 16-1	NM_128449	*Arabidopsis thaliana*	Involved in PORA precursor import	1
SEC12-like protein 2	NM_126208	*Arabidopsis thaliana*	Required for the formation or budding of transport vesicles from the ER	2
Glucomannan 4-beta-mannosyltransferase 2	NM_122180	*Arabidopsis thaliana*	Possesses glucomannan synthase and mannan synthase activities in vitro	1
FCS-Like Zinc finger 6	NM_106451	*Arabidopsis thaliana*	May act as an adapter to facilitate the interaction of SnRK1 complex with effector proteins	1
Ureide permease 5	NM_202186	*Arabidopsis thaliana*	Proton-coupled transporter that transports a wide spectrum of oxo derivatives of heterocyclic nitrogen compounds	1
Protein RALF-like 33	NM_117672	*Arabidopsis thaliana*	Mediates a rapid alkalinization of extracellular space by mediating a transient increase in the cytoplasmic Ca^{2+} concentration leading to a calcium-dependent signaling event	1
Glutamate receptor 2.8	NM_128468	*Arabidopsis thaliana*	May be involved in light-signal transduction and calcium homeostasis	1

photosystem II subunit R	NM_106555	*Arabidopsis thaliana*	Associated with the oxygen-evolving complex of photosystem II.	2
Disease resistance protein	NM_105280	*Arabidopsis thaliana*	Act as a partner in modulating RPS5-activated immune signaling	1
Probable magnesium transporter NIPA6	NM_127682	*Arabidopsis thaliana*	Acts as an Mg^{2+} transporter.	1
Ribulose bisphosphate carboxylase small subunit	NM_001125859	*Arabidopsis thaliana*	Functions to yield sufficient Rubisco content for leaf photosynthetic capacity	3
Ribulose bisphosphate carboxylase small subunit 1B	NM_001344249	*Arabidopsis thaliana*	Functions to yield sufficient Rubisco content for leaf photosynthetic capacity	1
Vacuolar protein sorting 55 (VPS55) family protein	NM_111986	*Arabidopsis thaliana*	Involved in late endosome to vacuole transport via multivesicular body sorting pathway	1
Reticulon-like protein B8	Niben101Scf12321g01024.1	*Nicotiana benthamiana* NM_180221	Participate in *A. tumefaciens* infection	1
Cysteine proteinase inhibitor 6	Niben101Scf12983g01058.1	*Nicotiana benthamiana* NM_180239	A specific inhibitor of cysteine proteinases. Probably involved in the regulation of endogenous processes and in defense against pests and pathogens	1
Calcium-binding protein 2	Niben101Scf07060g03008.1	*Nicotiana benthamiana* ***NM_104200***	Possesses sodium/calcium exchanger activity when expressed in a heterologous mammalian CHO-K1 cell system	2
Nucleotide-sugar transporter family protein	Niben101Scf03832g02016.1	*Nicotiana benthamiana* NM_119391	Involved in sugar transport.	1
Amino acid permease 6	Niben101Scf11899g00015.1	*Nicotiana benthamiana* NM_124341	Involved in the uptake of amino acids diffusing out of the xylem tracheids into the xylem parenchyma.	1
Calcium-binding EF-hand family protein	Niben101Scf02320g00018.1	*Nicotiana benthamiana* NM_104558	Mediates calcium uptake into mitochondria.	3
NAC domain-containing protein 86	Niben101Scf00308g01003.1	*Nicotiana benthamiana* NM_121732	Transcription factor directing sieve element enucleation and cytosol degradation	1
ATP synthase subunit b'	Niben101Scf04786g01004.1	*Nicotiana benthamiana* NM_119378	F1F0 ATP synthase produces ATP from ADP in the presence of a proton or sodium gradient.	1
Xyloglucan endotransglucosylase/hydrolase protein A	Niben101Scf00369g05011.1	*Nicotiana benthamiana* NM_121390	Catalyzes xyloglucan endohydrolysis and/or endotransglycosylation	1
Signal peptide peptidase-like 4	Niben101Scf08817g01012.1	*Nicotiana benthamiana* NM_100047	Cleaves type II membrane signal peptides in the hydrophobic plane of the membrane	1
Bax inhibitor 1	Niben101Scf02705g01020.1	*Nicotiana benthamiana* NM_124083	Involved in methyl jasmonate-induced leaf senescence through	2

			regulating cytoplasmic calcium level	
Chitinase	Niben101Scf02171g00007.1	*Nicotiana benthamiana* NM_115302	Probably involved in hypersensitive reaction upon *Xanthomonas campestris* infection.	2
Protein NRT1/ PTR FAMILY 2.13	Niben101Scf05189g00002.1	*Nicotiana benthamiana* NM_105655	Involved in phloem loading and nitrate remobilization from the older leaves to other tissues.	4
Photosystem II 22 kDa protein	Niben101Scf05304g05008.1	*Nicotiana benthamiana* NM_001333231	Plays an important role in non-photochemical quenching.	1
Kunitz trypsin inhibitor 1	Niben101Scf06424g01007.1	*Nicotiana benthamiana* NM_105985	Involved in modulating programmed cell death in plant-pathogen interactions.	1
Vesicle-associated membrane protein-associated protein A	Niben101Scf03599g01016.1	*Nicotiana benthamiana* NM_130077	Vesicle-associated protein that binds the oxysterol-binding protein ORP3A and allows its targeting to the ER.	1
Rhomboid-like protease 2	Niben101Scf09372g04009.1	*Nicotiana benthamiana* NM_123212	Probable rhomboid-type serine protease that catalyzes intramembrane proteolysis.	1
Protein of unknown function	Niben101Scf03154g04006.1	*Nicotiana benthamiana* NM_121285	May play a role in leaf development.	1
Tubby-like F-box protein 1	Niben101Scf07272g01001.1	*Nicotiana benthamiana* NM_103489	Involved in regulation of transcription, and response to fungus.	1
Unknown protein	Niben101Scf01464g00003.1	*Nicotiana benthamiana* NM_114357	Catalyzes the key reaction of chlorophyll catabolism	2
V-type proton ATPase 16 kDa proteolipid subunit	Niben101Scf10919g00001.1	*Nicotiana benthamiana* NM_119638	Proton-conducting pore forming subunit of the membrane integral V0 complex of vacuolar ATPase.	3
Galact isoform 1	Niben101Scf11909g00001.1	*Nicotiana benthamiana* NM_105805	It is involved in early seed development and nuclear morphology.	1
PRA1 (Prenylated rab acceptor) family protein	Niben101Scf09321g02005.1	*Nicotiana benthamiana* NM_112222	May be involved in both secretory and endocytic intracellular trafficking in the endosomal/prevacuolar compartments.	1
Monocopper oxidase-like protein SKU5	Niben101Scf09555g01004.1	*Nicotiana benthamiana* NM_117312	Involved in directional growth processes, possibly by participating in cell wall expansion.	2
TETRASPANIN8	Niben101Scf05110g00007.1	*Nicotiana benthamiana* NM_179714	Involved in cross-kingdom RNA interference	1
Calcium-transporting ATPase	Niben101Scf04852g01008.1	*Nicotiana benthamiana* NM_124680	Involved in calcium ion transmembrane transport	1
CURVATURE THYLAKOID 1B	Niben101Scf02026g02002.1	*Nicotiana benthamiana* NM_130248	Determines thylakoid architecture by inducing membrane curvature	1
Plasma membrane ATPase 1	Niben101Scf03979g02010.1	*Nicotiana benthamiana* NM_001203941	Pumps protons out of the cell, generating a proton gradient that	1

			drives the active transport of nutrients by proton symport	
Chitinase 8	Niben101Scf02041g00002.1	*Nicotiana benthamiana* NM_112085	Defense against chitin-containing fungal pathogens.	1
Photosystem II 10 kDa polypeptide	Niben101Scf02044g06014.1	*Nicotiana benthamiana* NM_106555	Associated with the oxygen-evolving complex of photosystem II.	2
Pyruvate kinase family protein	Niben101Scf02502g16008.1	*Nicotiana benthamiana* NM_115159	Involved in synthesizing pyruvate from D-glyceraldehyde 3-phosphate	1
Squalene synthase	Niben101Scf04664g02013.1	*Nicotiana benthamiana* NM_119630	Catalyzes the first pathway-specific reaction of the sterol branch of the isoprenoid pathway.	1
Non-specific lipid-transfer protein 1	Niben101Scf29144g00011.1	*Nicotiana benthamiana* NM_001202913	May play a role in wax or cutin deposition in the cell walls of expanding epidermal cells and certain secretory tissues.	3
Homeobox protein knotted-1-like 3	Niben101Scf09454g00008.1	*Nicotiana benthamiana* NM_121144	DNA-binding transcription factor activity.	1
Transmembrane 9 superfamily member 7	Niben101Scf02363g00025.1	*Nicotiana benthamiana* NM_112228	May play a role as an effector of cellular copper homeostasis.	1
Peroxisomal membrane protein PEX16	Niben101Scf03676g04011.1	*Nicotiana benthamiana* NM_130132	Involved in the formation of peroxisomes, lipid bodies, and protein bodies.	1
Universal stress protein A-like protein	Niben101Scf10205g00001.1	*Nicotiana benthamiana* NM_115259	Functions as a molecular chaperone under heat shock and oxidative stress conditions.	1
Calcium-dependent lipid-binding (CaLB domain) family protein	Niben101Scf00107g02001.1	*Nicotiana benthamiana* NM_119578	Involved in protein binding	1
PGR5-like B	Niben101Scf01729g01002.1	*Nicotiana benthamiana* NM_117266	Involved in cyclic electron flow around photosystem I.	1
Aquaporin-like superfamily protein	Niben101Scf03772g03005.1	*Nicotiana benthamiana* NM_117838	Facilitate the transport of water and small neutral solutes across cell membranes.	1
Phosphatidylinositol-glycan biosynthesis class F protein	Niben101Scf09928g01002.1	*Nicotiana benthamiana* NM_101472	This protein is involved in the pathway glycosylphosphatidylinositol-anchor biosynthesis, which is part of Glycolipid biosynthesis.	1
Glyceraldehyde-3-phosphate dehydrogenase	Niben101Scf08544g00004.1	*Nicotiana benthamiana* NM_101214	Key enzyme in glycolysis catalyzes the first step of the pathway by converting D-glyceraldehyde 3-phosphate into 3-phospho-D-glyceroyl phosphate.	1
S-adenosylmethionine synthase 1-like (METK1)	NM_001288680	*Solanum tuberosum* NM_100131	Catalyzes the formation of S-adenosylmethionine from methionine and ATP.	2
Autophagy-related protein 8f	XM_006355410	*Solanum tuberosum* NM_117751	Ubiquitin-like modifier involved in autophagosomes formation.	1

Cysteine proteinase inhibitor A-like	XM_006362671	*Solanum tuberosum* NM_180239	A specific inhibitor of cysteine proteinases. Probably involved in the regulation of endogenous processes and in defense against pests and pathogens.	1
Calmodulin-2/4-like	XM_006367914	*Solanum tuberosum* NM_115539	Calmodulin mediates the control of a large number of enzymes, ion channels, and other proteins by Ca^{2+}	1
Probable glutathione S-transferase	XM_006366714	*Solanum tuberosum* NM_106485	Catalyzes the glutathionylation of 12-oxophytodienoate	1
Ribulose bisphosphate carboxylase small subunit 2C	XM_006363049	*Solanum tuberosum* NM_001344249	The basic functional RuBisCO is composed of a large chain homodimer in a 'head-to-tail' conformation	6
Glucose-6-phosphate/phosphate translocator 2	XM_006367218	*Solanum tuberosum* NM_104862	Glucose 6-phosphate transporter	1
Catalase isozyme 1	NM_001287934	*Solanum tuberosum* NM_101914	Protect cells from the toxic effects of hydrogen peroxide	1
Aquaporin PIP2-7-like	NM_001318604	*Solanum tuberosum* NM_119676	A water channel is required to facilitate the transport of water across the cell membrane	1
Photosystem II 10 kDa polypeptide	JX683432	*Solanum tuberosum* NM_106555	Associated with the oxygen-evolving complex of photosystem II.	2
L-ascorbate peroxidase 3	XM_006340696	*Solanum tuberosum* NM_119666	Plays a key role in hydrogen peroxide removal.	1
Probable receptor-like protein kinase	XM_006339552	*Solanum tuberosum* NM_122306		1
Triose phosphate/phosphate translocator	NM_001287896	*Solanum tuberosum* NM_001085251	Mediates the export of fixed carbons from the chloroplasts into the cytosol in the form of triose phosphates.	1
Zinc finger BED domain-containing protein DAYSLEEPER-like	XM_015307756	*Solanum tuberosum* NM_114084	Transposase-like protein that is essential for plant growth and development	1
Pectinmethylesterase inhibitor	KJ788180	*Solanum tuberosum* NM_104955	Regulates de-methylesterification of pectins in roots, and affects root growth.	1
NAC domain-containing protein 78-like	XM_015315417	*Solanum tuberosum* NM_111885	Transcriptional activator activated by proteolytic cleavage through regulated intramembrane proteolysis	1
Steroid 5-alpha-reductase DET2	XM_006363009	*Solanum tuberosum* NM_129361	Involved in a reduction step in the biosynthesis of the plant steroid, brassinolide	1
R3H domain-containing protein 2	XM_006364719	*Solanum tuberosum* NM_129661	Single-stranded nucleic acid binding R3H protein	1

Photosystem I reaction center subunit XI	XM_006366755	*Solanum tuberosum* NM_117349	Encodes subunit L of photosystem I reaction center	1
Vesicle-associated membrane protein 714	XM_006361020	*Solanum tuberosum* NM_122141	Involved in the targeting and/or fusion of transport vesicles to their target membrane	1
Eukaryotic initiation factor 5A5-like	DQ222495	*Solanum tuberosum* NM_105608	Involved in supporting growth and plays a regulatory role in the response to sub-lethal osmotic and nutrient stress	1
Plasma membrane ATPase 4-like	NM_001287919	*Solanum tuberosum* NM_001203941	Pumps protons out of the cell, generating a proton gradient that drives the active transport of nutrients by proton symport	1
Cytochrome b5 isoform E	XM_015310118	*Solanum tuberosum* NM_124736	Functions as an electron carrier for several membrane bound oxygenases, including fatty acid desaturases	1
Respiratory burst oxidase homolog protein C	NM_001288524	*Solanum tuberosum* NM_124485	Required for H_2O_2 production in response to K^+ deficiency and for the generation of reactive oxygen species that regulate cell expansion through the activation of Ca^{2+} channels	1
Protease inhibitor-related protein	EU849681	*Solanum tuberosum*	defense response	2
2-methyl-6-phytyl-1,4-hydroquinone methyltransferase	XM_006355979	*Solanum tuberosum* NM_116206	Involved in a key methylation step in both tocopherols and plastoquinone synthesis	1
Exocyst complex component SEC3A	XM_006342542	*Solanum tuberosum* NM_001198234	Involved in tethering vesicles to the plasma membrane during regulated or polarized secretion. It binds phosphoinositide lipids.	2
Heat shock 70 kDa protein 2-like	XM_006340980	*Solanum tuberosum* NM_120327	Facilitate the folding of de novo synthesized proteins, assist translocation of precursor proteins into organelles, and are responsible for the degradation of damaged protein under stress conditions.	1
Ribulose-1,5-bisphosphate carboxylase/oxygenase small subunit	EU294360	*Solanum tuberosum* NM_001344249	The basic functional RuBisCO is composed of a large chain homodimer in a 'head-to-tail' conformation.	1
Major intrinsic protein 2	DQ235182	*Solanum tuberosum* NM_115202	A water channel is required to facilitate the transport of water across the cell membrane	1
Ubiquitin-conjugating enzyme E2 34-like	XM_006342828	*Solanum tuberosum* NM_001332287	Accepts the ubiquitin from the E1 complex and catalyzes its covalent attachment to other proteins.	1
Ubiquitin receptor RAD23b isoform X1	XM_006341369	*Solanum tuberosum* NM_001198510	Involved in nucleotide excision repair.	1

ABC transporter C family member 12-like isoform X9	XM_015312116	*Solanum tuberosum* NM_001332898	Pump for glutathione S-conjugates.	1
LysM domain receptor-like kinase 4	XM_006338210	*Solanum tuberosum* NM_127940	Involved in the resistance to the pathogenic fungus *Alternaria brassicicola* and to the bacterial pathogen *Pseudomonas syringae* pv tomato DC3000	1
Polyadenylate-binding protein-interacting protein 9-like	XM_006359958	*Solanum tuberosum* NM_112305		1
Cathepsin B-like	XM_006362540	*Solanum tuberosum* NM_116392	Plays a central role in plant programmed cell death	1
Novel plant SNARE 13-like	XM_006349802	*Solanum tuberosum* NM_112623	Vesicle trafficking protein that functions in the secretory pathway.	1
Importin-4	XM_006349312	*Solanum tuberosum* NM_118900	Nuclear import receptor for GRF-interacting factors, roles in ovule development.	1
Probable zinc metallopeptidase EGY3	XM_006346219	*Solanum tuberosum* NM_101650	May be involved in chloroplast development.	1
Protein SDE2 homolog	XM_006339637	*Solanum tuberosum* NM_001340294		1
Plastidic ATP/ADP-transporter	NM_001287865	*Solanum tuberosum* NM_106679	May function as an ATP importer.	1
ECERIFERUM 1-like	XM_006361451	*Solanum tuberosum* NM_001197961	Involved in epicuticular wax biosynthesis and pollen fertility.	1
Chitinase	STU02606	*Solanum tuberosum* NM_112085	Defense against chitin-containing fungal pathogens.	1

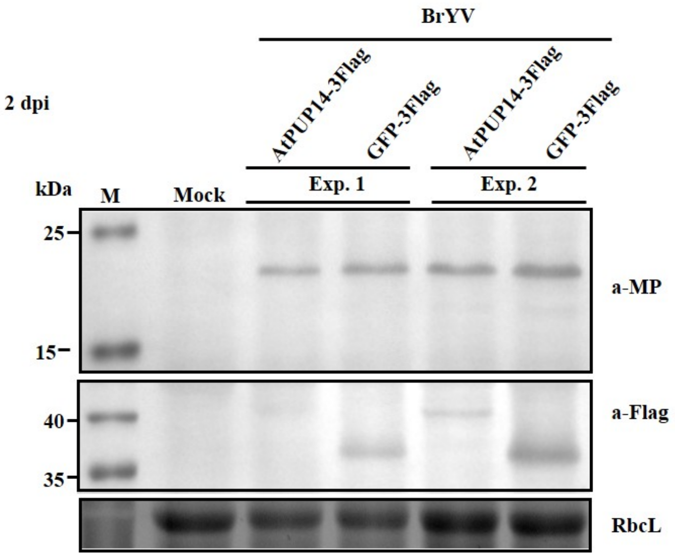

Figure S1. Overexpression of AtPUP14 decreased local BrYV accumulation level in inoculated leaves.
